# Phagentherapie in Deutschland – Update 2025

**DOI:** 10.1007/s00103-025-04063-z

**Published:** 2025-05-08

**Authors:** Christian Willy, Felix Bröcker

**Affiliations:** 1 Abteilung Unfall- & Orthopädische Chirurgie, Septisch-Rekonstruktive Chirurgie, Forschungs- und Behandlungszentrum Septische Defektwunden, Bundeswehrkrankenhaus Berlin, Scharnhorststraße 13, 10115 Berlin, Deutschland; 2 Idorsia (Berlin) Pharmaceuticals GmbH, Berlin, Deutschland

**Keywords:** Phagentherapie, Phagenherstellung, Forschungsprojekte, Phagenbank, Deutschland, Phage therapy, Phage production, Research projects, Phage bank, Germany

## Abstract

Die Phagentherapie ist ein vielversprechender Ansatz, um die Antibiotikaresistenzkrise weltweit und in Deutschland zu bekämpfen. Nach anfänglich weitverbreiteter Anwendung in Deutschland in den 1930er- bis 1960er-Jahren sank jedoch die Zahl der jährlich mit Phagen behandelten Patienten in den folgenden Jahrzehnten auf derzeit unter 100. Es wird ein Überblick über die aktuelle Situation der Phagentherapie in Deutschland und die sie unterstützende translationale Forschung gegeben. Berücksichtigte Aspekte sind hierbei: Phagenproduktion, Phagenbanken, klinische Phagenanwendung und laufende translationale Forschungsprojekte in Deutschland.

Es fehlt an Phagen für den klinischen Einsatz. Die Phagentherapie wird nur bei einer begrenzten Anzahl von Patienten in wenigen Kliniken als individueller Behandlungsversuch angewendet. Es gibt mehrere Phagenbanken, die aufgrund unterschiedlicher institutioneller Hintergründe und Richtlinien nicht auf vergleichbare Weise arbeiten und die Phagenbestände *lediglich im Rahmen wissenschaftlicher Projekte* austauschen. Projekte zur Grundlagen- und translationalen Phagenforschung haben sich in Deutschland erheblich vermehrt. Die dringendste Änderung scheint die Erweiterung der Phagenproduktionskapazitäten zu sein.

Es gibt im medizinischen Bereich immer noch keine hochwertige Evidenz zum klinischen Erfolg der Phagentherapie im Rahmen von randomisierten kontrollierten klinischen Studien. In enger Abstimmung mit den Regulierungsbehörden erscheint es sinnvoll, dringendst einigen Zentren die Behandlung von Patienten nach dem belgischen Modell zu ermöglichen.

## Abstract (en)

Phage therapy is a promising approach to combat the antibiotic resistance crisis worldwide and in Germany. However, after widespread use in Germany from the 1930s to the 1960s, the number of patients treated with phages each year dropped to under a hundred in the following decades. An overview of the current situation of phage therapy in Germany and the translational research supporting it is provided. Aspects taken into account here are phage production, phage banks, clinical phage application, and ongoing translational research projects in Germany.

There is a lack of phages for clinical use. Phage therapy is only used in a limited number of patients in a few clinics as an individual treatment attempt. There are several phage banks that, due to different institutional backgrounds and guidelines, do not work in a comparable way and only exchange phage stocks in the context of scientific projects. Projects in basic and translational phage research have increased considerably in Germany. The most urgent change seems to be the expansion of phage production capacities.

There is still no high-quality evidence on the clinical success of phage therapy in the medical field by means of randomized controlled clinical trials. In close consultation with the regulatory authorities, it seems sensible to urgently enable a few centers to treat patients according to the Belgian model.

## Einleitung

Die Phagentherapie hat ihren Ursprung im frühen 20. Jahrhundert. Sie wurde 1917 von Félix d’Hérelle entdeckt. Er beobachtete, wie Phagen in der Lage waren, bakterielle Infektionen zu eliminieren. Seine bahnbrechende Arbeit führte 1919 zur ersten dokumentierten erfolgreichen Phagentherapie einer bakteriellen Infektion. Er behandelte einen 12-jährigen Jungen, der an schwerer bakterieller Dysenterie litt, mit einer Phagenlösung im Hôpital Necker-Enfants Malades, einem seit 1778 bis heute bestehenden Kinderkrankenhaus in Paris. Nach der Verabreichung erholte sich der Patient schnell und vollständig. Dieser Erfolg markierte den Beginn der Phagentherapie und legte den Grundstein für weitere Forschungen und Anwendungen in diesem Bereich, vor allem in Frankreich und Georgien, wo in Tiflis (heute Tbilisi) der georgische Mikrobiologe George Eliava, ein Freund von d’Hérelle, das nach ihm benannte Institut 1923 gründete. Das Eliava-Institut spielt bis heute eine bedeutende Rolle in der Phagenforschung und -therapie.

In den 1930er- und 1940er-Jahren erlebte die Phagentherapie in Europa eine Blütezeit. Unternehmen in Deutschland (Behring-Werke) und den USA (Eli Lilly) stellten Phagen her. Dysenterie-Polyfagin® gegen Shigella-Infektionen war das erste Phagenpräparat, das die Behringwerke bei Marburg/Lahn im Juli 1939 auf den Markt brachten. Kurze Zeit später wurde mit Typhus-Polyfagin® ein weiteres Präparat gegen Salmonelleninfektionen hergestellt. Viele deutsche Soldaten wurden in Afrika mit Phagen behandelt.

Tatsächlich wurden positive Therapieergebnisse, aber auch – nach anfänglicher Euphorie – entsprechende Misserfolge gemeldet. Es wird vermutet, dass diese in der Regel darauf zurückzuführen waren, dass den Klinikern die Wirtsspezifität der Phagen und wichtige Anwendungsparameter nicht bekannt waren und wahrscheinlich ungeeignete Phagen verwendet wurden (temperente Phagen, die ihr Erbgut (Prophage) ins Genom der Wirtszelle einbauen und sie nicht zerstören). Darüber hinaus beeinträchtigten prozessbedingte Verunreinigungen und die mangelnde Stabilität der Phagenlösungen die Produktionsqualität. Daraus resultierende Misserfolge, die Markteinführung von Antibiotika für den klinischen Bereich (Sulfonamide 1935, Penicillin 1942, Streptomycin 1943, Tetracycline 1948) sowie Sprachbarrieren und der in der Nachkriegszeit entstandene politische Graben führten zum Nachlassen des Interesses an der Phagentherapie. Während in Westeuropa die Phagentherapie fast vollständig in Vergessenheit geriet, blieb sie in Osteuropa, insbesondere in der damaligen Sowjetunion, populär.

Noch in den 1960er-Jahren stand in der Deutschen Demokratischen Republik (DDR) ein gebrauchsfertiges Phagenpräparat, Intestolysin®, zur Verfügung (gegen Typhus, Paratyphus, Dysenterie und pathogene *Escherichia coli*, hergestellt vom Institut für Seuchenbekämpfung in Berlin-Weißensee). Zeitweise mussten enorme Phagenmengen produziert (11.000 l pro Woche, Titer bis zu 10^12^ Plaque-forming Units (PFU)/mL) und in epidemischen Situationen eine Phagenprophylaxe der Bevölkerung realisiert werden (z. B. vom 26.04.1962 bis zum 14.06.1962 für insgesamt 174.906 Menschen; [1]).

Dann Ende der 1970er-Jahre wurden in der ENDO-Klinik Hamburg über einen Zeitraum von mindestens 5 Jahren Phagen zur Behandlung periprothetischer Infektionen eingesetzt (*n* = 54, *Follow-up *> 1 Jahr bei *n* = 17, 14/17 rezidivfrei). In der Folgezeit wurden an der Medizinischen Hochschule Hannover, im Helios Klinikum Hildesheim und in den Krankenhäusern Chemnitz und Tirschenreuth im Bereich unfallchirurgisch/orthopädische Infektionen Phagen eingesetzt.

Im Oktober 2017, 100 Jahre nach der Entdeckung der Phagen, fand das erste deutsche Phagen-Symposium an der Universität Hohenheim in Stuttgart statt.

Heute erlebt die Phagentherapie eine Renaissance in Europa. Sie wird zunehmend als *individualisierte Therapieform und ergänzende Option zur Antibiotikagabe betrachtet*. Vor allem in Belgien und Frankreich, seit November 2024 auch in Portugal, gibt es neue Therapiezentren, die sich auf die Entwicklung personalisierter Phagenbehandlungen spezialisiert haben. Phagenherstellungen erfolgen zudem in Slowenien, Polen und Tschechien. Trotz regulatorischer Hürden wächst auch in Deutschland die Hoffnung, die Phagentherapie als festen Bestandteil der modernen Medizin zu etablieren. Vor diesem Hintergrund soll im Folgenden die aktuelle Situation der Phagentherapie in Deutschland vor allem aus der Perspektive von Klinikern dargestellt werden.

Seit 2017 hatte sich in Deutschland ein interdisziplinäres Netzwerk aus Ärzten, Pharmazeuten, Mikrobiologen, Bioingenieuren, Bioinformatikern – alle mit einem patientenorientierten Fokus – gebildet [2]. Letztlich führte das gemeinsame Interesse für Phagen in Wissenschaft und klinischer Anwendung im Juli 2022 zur Gründung des Translationalen Phagen-Netzwerkes „DZIF Translational Phage-Netzwerk“ (kurz: DZIF TransPhage-Net) des Deutschen Zentrums für Infektionsforschung (DZIF; siehe Infobox).

Aktuell wird im Rahmen der DZIF-Arbeit mit der Koordination durch die Deutsche Gesellschaft für Infektiologie [3] anhand eines Leitlinienteams bestehend aus 15 Fachgesellschaften, 15 nationalen und internationalen Expertinnen und Experten, inkl. Vertretung des Bundesinstituts für Arzneimittel und Medizinprodukte (BfArM) und des Paul-Ehrlich-Instituts (PEI), als auch 2 Patientenvertretungen eine AWMF-S2k-Leitlinie (Arbeitsgemeinschaft der Wissenschaftlichen Medizinischen Fachgesellschaften; Registernummer: 092-003) zur Phagentherapie erarbeitet (voraussichtliches Ende der Leitlinienerstellungsarbeit Mitte 2025). Die konsensbasierten Empfehlungen der Leitlinie beschreiben erstmals umfassend die allgemeinen Prinzipien, Anforderungen an die Infrastruktur, Herstellung und Qualitätskontrollen und die Anwendung sowie zentrale Forschungsfragen für eine personalisierte Phagentherapie in Deutschland.

Um das Ausmaß der klinischen Anwendung von Phagen in Deutschland, die aktuellen Aktivitäten zur Herstellung von Phagen für diesen Zweck, den Status der Phagenbanken mit ggf. vorrätigen Phagenlösungen und das Ausmaß der aktuellen, vor allem translationalen Forschungsansätze zusammenzustellen, wurde auch innerhalb des DZIF TransPhage-Nets nach aktiven Gruppen gesucht und die gesammelten Daten mit aktuellen Informationen aus der Literatur abgeglichen [2].

## Die klinische Anwendung von Phagen in Deutschland heute

Phagen zur therapeutischen Anwendung beim Menschen sind biologische Arzneimittel und derzeit in Deutschland nicht zugelassen. Dennoch kann eine Therapie als „individueller Behandlungsversuch“, unter der Verantwortung des behandelnden Arztes im Rahmen der Therapiefreiheit und mit Zustimmung des Patienten erfolgen, wenn ein „ungedeckter medizinischer Bedarf“ gemäß Artikel 37 der Deklaration von Helsinki des Weltärztebundes vorliegt (d. h. unbewiesene Interventionen in der klinischen Praxis) und der behandelnde Arzt aufgrund wissenschaftlicher Erkenntnisse einen Nutzen des Phageneinsatzes für den Patienten vermutet. Diese Situation unterscheidet sich von der seit 2016 formell bestehenden Regelung in Belgien (*Belgian Magistral Phage Medicine Strategy), *mit der sich nicht auf diesen ethischen Rahmen abgestützt werden muss, Magistralrezepturen nicht nach Guter Herstellungspraxis (GMP) hergestellt sein müssen, jedoch Herstellungsschritte und mit den Bestimmungen der nationalen (belgischen) Monografie übereinstimmen müssen. Vorteil ist zudem, dass die Patientendaten in klinischen Studien verwendet werden dürfen, was unter dem Anwendungsschutzschirm des Artikels 37 nicht möglich ist.

Auf der Basis der bislang 4 durchgeführten randomisierten kontrollierten Studien (RCT, Evidenzklasse 1b) besteht kein Wirksamkeitsbeweis im strengen Sinne. Ursachen hierfür sind zahlreiche methodische Limitationen wie geringer Phagentiter der verabreichten Phagenlösung, zu geringer Anteil effizienter Phagen in der Phagenlösung, Phagendestruktion bei Magen-Darm-Passage, da keine Neutralisierung der Magensäure, keine Erstellung von Phagogrammen zur Sensibilitätstestung vor Applikation. Eine zukunftsweisende Beurteilung des Potenzials der Phagentherapie mit Studien sehr hoher Evidenz (RCT) war deshalb bisher nicht möglich.

Dennoch zeigt die Literatur eindeutige Hinweise hoher Evidenz für eine Wirkung der Phagentherapie (Level 1c in der Klassifikation der evidenzbasierten Medizin (EbM; [4]) als „All-or-None“-Prinzip eine Sonderform innerhalb der höchsten Evidenzstufe (Level I)). Sie bezieht sich nicht auf klassische RCTs (Level 1a und 1b), sondern auf eine besondere klinische Beobachtung: Wenn vor der Intervention alle Patienten an einer bestimmten Erkrankung starben (oder ein bestimmtes unerwünschtes Ereignis immer auftrat), nach Einführung der Intervention jedoch einige Patienten überleben oder das Ereignis nicht mehr eintritt). Und aktuelle systematische Übersichtsarbeiten deuten an, dass der Phageneinsatz in ca. 80 % zu einer klinischen Heilung, in ca. 10 % zu einer Besserung sowie in ca. 10 % zu keiner Besserung führt. Eine zentrale nationale oder internationale Datenbank (Register), in der die Behandlungsdaten der wenigen in Deutschland therapierten Patienten dokumentiert werden, besteht nicht. Allerdings zeigte eine in Belgien aktuell durchgeführte Registerstudie bei den ersten 100 eingeschlossenen Behandlungen in 77,2 % der Fälle eine klinische Verbesserung und eine komplette Erregereradikation von 61,3 % [5].

Für eine Änderung des Zulassungsrechtsrahmens ist es allerdings erforderlich, die Wirksamkeit von Phagen umfangreicher nachzuweisen, um den Besonderheiten der Phagenprodukte Rechnung zu tragen. Hierfür sind weitere randomisiert kontrollierte Studien (RCT) unabdingbar und eine Registerstudie hilfreich.

Im Folgenden werden Anwendungsbeispiele für Phagentherapie an 5 deutschen Kliniken dargestellt.

### Berlin.

Am Bundeswehrkrankenhaus Berlin werden seit 2016 zunächst Patienten mit Phagen aus Georgien behandelt, zum Teil in Zusammenarbeit mit dem Labor für molekulare und zelluläre Technologie (LabMCT) am Königin-Astrid-Militärkrankenhaus in Brüssel (Belgien) und der Charité Universitätsmedizin Berlin, Zentrum für Muskuloskelettale Chirurgie. Im Rahmen des Forschungsprojekts *PhagoFlow*, gefördert durch den Innovationsfonds des Gemeinsamen Bundesausschusses (G-BA), werden seit 2023 Patienten mit Infektionen durch *Pseudomonas aeruginosa* mit magistralen Phagenpräparaten behandelt (www.phagoflow.de).

### Hannover.

An der Medizinischen Hochschule Hannover ist die Phagentherapie in der Klinik für Herz‑, Thorax‑, Transplantations- und Gefäßchirurgie angesiedelt. Das Portfolio der Abteilung umfasst 33 Fälle von personalisierter Phagentherapie bei schwerkranken Patienten seit 2015, von denen 31 erfolgreich waren. Die hohe klinische Erfolgsrate (> 90 %) ist auf eine Kombination aus modernen Prinzipien der erlaubnisfreien Präparation von meist selbstisolierten Phagen, einem interdisziplinären Ansatz zur Verabreichung von Phagen und einer optimalen begleitenden konventionellen Behandlung, einschließlich der Antibiotikatherapie, zurückzuführen [6, 7]. Im Rahmen eines akademischen klinischen Zentrums (Nationales Phagenzentrum der Medizinischen Hochschule Hannover) sind die Auswahl gut charakterisierter Phagen unter Verwendung etablierter Phagogrammmethoden und die Herstellung ausgewählter Phagen in den Laboreinrichtungen der Klinik gewährleistet.

### Regensburg.

An der Klinik für Unfallchirurgie des Universitätsklinikums Regensburg wird in Zusammenarbeit mit dem Institut für Mikrobiologie und Hygiene (Phage Center Regensburg) die Phagentherapie seit Oktober 2022 auch mittels innovativer Phagenträgersubstanzen durchgeführt (Hydrogel [8]). Die Klinik nahm an der französischen PhagoDAIR-Studie teil, einer randomisierten, nicht vergleichenden, doppelblinden klinischen Studie der Phase I/II bei Patienten mit periprothetischen Gelenkinfektionen durch *Staphylococcus aureus* am Knie oder an der Hüfte, bei denen eine Indikation für Debridement, Antibiotika- und Implantaterhalt (DAIR) in Kombination mit einer suppressiven Antibiotikatherapie besteht (ClinicalTrials.gov Identifier: NCT05369104).

### Rostock.

Die Phagentherapie wird auch in der Klinik für Allgemein‑, Viszeral‑, Thorax‑, Gefäß- und Transplantationschirurgie der Universitätsmedizin Rostock durchgeführt. Im Bereich der Gefäßchirurgie sind es vor allem Patch- oder Bypass-assoziierte Infektionen in der Leistengegend, gefolgt von akut lebensbedrohlichen Aortenprotheseninfektionen, die mit Phagentherapie behandelt werden. Durch die Fokussierung auf die individualisierte Phagentherapie wurde eine gute Erfolgsquote erreicht. Aktuell ist die Eigenproduktion vor Ort geplant. Aktuelle und geplante Forschungsprojekte konzentrieren sich auf die Prüfung der Thermostabilität von Phagen in Knochenzement, die topische Phagenapplikation bei infizierten Endo-Exo-Systemen am Stoma und die Planung der Studie „Bacteriophage related treatment of chronicly infected Drive-Line/LVAD Systems: a randomised controlled multicentre trial“ (über Antrag bei der Deutschen Forschungsgemeinschaft – DFG) in Zusammenarbeit mit dem Herzzentrum Leipzig, Deutschland.

### Halle an der Saale.

An der BG-Klinik in Halle wurde in den letzten 12 Monaten für 245 Patienten die Testung der Möglichkeit einer Phagenanwendung und bei 15 Patienten eine Phagentherapie durchgeführt. Behandelt wurden vornehmlich periprothetische Infekte, chronische Knocheninfektzustände sowie ebenso vereinzelt Weichteilinfekte und Komplikationen des diabetischen Fußsyndroms. Die Phagen wurden oral und mittels Drainagen topisch appliziert. In aller Regel wurden fertige Phagenmixturen aus Georgien (Eliava, Phagebiolab Tiflis) verwendet.

## Produktion von Phagen

Das Fraunhofer-Institut für Toxikologie und Experimentelle Medizin (ITEM), Braunschweig, entwickelte einen plattformähnlichen Herstellungsprozess für natürliche Phagen als pharmazeutische Wirkstoffe (Active Pharmaceutical Ingredient, API) unter Verwendung von Wirtsbakterien unter GMP-konformen Bedingungen. Dazu wurden zunächst die Ausgangsmaterialien *Master Cell Banks* (MCB) und *Master Phage Stocks* (MPS) GMP-gerecht hergestellt. Die Phagenwirkstoffproduktion begann mit der Kultivierung (bis zu 10 L) des Produktionsstammes (MCB) und der Infektion mit Phagen (MPS) und anschließender Zelllyse. Das Lysat mit den Phagen wurde zunächst durch Tiefenfiltration abgetrennt (Zellfragmentreduktion), in einem für die Chromatografie geeigneten Puffer erneut gepuffert (Diafiltration) und anschließend chromatografisch gereinigt, um die schädlichen Endotoxine (bakterielle Toxine, die bei der Phagenherstellung in Bakterien in das Phagenlysat gelangen) und Wirtszellproteine (potenziell schädliche Proteine der Bakterien im Phagenlysat) zu entfernen. Es folgte eine zweite Diafiltration mit einem physiologischen Puffer und eine 0,2 µm-Filtration zur Verminderung der bakteriellen Kontamination. Schließlich wurde der Phagenwirkstoff in die Primärverpackung abgefüllt. Die Herausforderung bei der Entwicklung des Herstellungsprozesses bestand darin, geeignete Bedingungen zu finden, die die höchsten Titer, Lysatvolumen und die Reduzierung von Endotoxinen und Wirtszellproteinen ermöglichen. Die Herstellungserlaubnis für die Phagenproduktion in Deutschland nach GMP war im August 2022 erteilt worden.

Eine alternative Methode zur Herstellung von Phagen, die Expression in einem *In-vitro*-System, wird derzeit von INVITRIS (invitris.com), einem Spin-off der Technischen Universität München, entwickelt. Bei der *In-vitro-* oder zellfreien Proteinsynthese („cell-free protein synthesis“*,* CFPS) wird die zelluläre Expressionsmaschinerie aus der Zelle, meist *E. coli*, isoliert und mit Metaboliten, Vorläufermolekülen und einer DNA-Vorlage kombiniert, um eine breite Palette von Produkten herzustellen. CFPS wurde zur Herstellung von *E.-coli*-Phagen wie T7, phiX174 und MS2 mit Titern von bis zu 10^12^–10^13^ PFU/mL verwendet [9]. Jüngste Verbesserungen des Systems ermöglichten die Expression von Phagen, die nicht zu *E. coli* gehören, einschließlich Phagen gegen klinisch relevante Krankheitserreger wie *Yersinia pestis* und sogar grampositive Bakterien wie *Bacillus subtilis* [10]. Die Produktion von therapeutischen Wirkstoffen in CFPS reduziert den Endotoxingehalt und die Verwendung eines gut charakterisierten Spenderstamms beseitigt Bedenken hinsichtlich einer Prophagenkontamination. CFPS-Reaktionen lassen sich leicht auf unterschiedliche Volumina skalieren. Die wirtsunabhängige Produktion von Phagen in einem einzigen System ermöglicht einen geringeren Zeit- und Materialaufwand für die personalisierte Phagenanwendung.

Das Herstellungsverfahren nach § 13 (2b) AMG (erlaubnisfreie Herstellung von Arzneimitteln) wird derzeit nur an der Medizinischen Hochschule Hannover (Klinik für Herz‑, Thorax‑, Transplantations- und Gefäßchirurgie) gemäß den Qualitäts- und Sicherheitsanforderungen für nachhaltige Phagentherapieprodukte durchgeführt. Für die Produktion werden ausschließlich streng lytische Phagen verwendet, die keine bekannten Gene für Integrasen, bakterielle Virulenz- und Antibiotikaresistenzfaktoren enthalten. Obwohl bei den Verbrauchsmaterialien für die Produktion bislang kein herkömmliches GMP-Verfahren angewandt wird, werden Nährmedien und Reagenzien verwendet, die entweder für die GMP-Produktion oder für den klinischen Einsatz bestimmt sind. Die Phagenamplifikation erfolgt nach der ursprünglichen Methode im kleinen Maßstab auf dichten Nährmedien, die stabile hohe Titer von Phagen ergeben hat und keine Optimierung des Produktionsprozesses erfordert. Nach der Amplifikation wird das Phagenlysat einer mehrstufigen Reinigung unterzogen, um nichtlysierte Zellen, Bestandteile des Nährmediums und Pyrogene zu entfernen, gefolgt von einer Kontrolle der Sterilität, des Phagentiters und der Identität sowie des Endotoxingehalts. Derzeit streben auch andere Kliniken in Deutschland (Berlin, Regensburg, Rostock) diese Form der Produktion an. Bei all diesen Herstellungsschritten wird den Vorgaben der European Pharmacopoeia gefolgt [11].

Auch am Helmholtz-Zentrum München und an der Technischen Universität München (TUM) soll vor dem Hintergrund zahlreicher Forschungsprojekte (siehe unten) eine GMP-Phagenherstellungsanlage eingerichtet werden, die schließlich den Beginn klinischer Studien (insbesondere gegen die ESKAPE-Bakterien (*Enterococcus faecium, S. aureus, Klebsiella pneumoniae, Acinetobacter baumannii, P. aeruginosa* und *Enterobacter *spp.)) erleichtern wird. Diese Anstrengungen münden dort in den aktuell erfolgenden Aufbau eines Bayerischen Kompetenzzentrums für Phagentherapie am Zentralinstitut für Infektionsprävention (ZIP) der TUM als Grundlage für einen baldigen Einsatz von Phagen in einem klinischen Umfeld.

## Status der Phagenbanken

Phagensammlungen (Phagenbanken) sind zu mannigfaltigen Zwecken in zahlreichen Laboratorien und Forschungsinstituten in Deutschland angelegt. Nachfolgend sind die Phagenbanken aufgeführt, deren Phagen unmittelbar für Phagentherapiestudien und -anwendungen eingesetzt werden. Auffallend ist, dass diese Phagenbanken aufgrund ihres unterschiedlichen institutionellen Hintergrunds und der Politik, an die sie gebunden sind (z. B. Bioressourcenzentrum oder Universität), nicht in vergleichbarer Weise arbeiten und ihre Phagenbestände i. d. R. nur für wissenschaftliche Projekte austauschen können. Auch gibt es keine vollständige Genomsequenzierung der jeweiligen Phagensammlungen. So ist es auch verständlich, dass eine vollständige und gemeinsame Datensammlung aller bioinformatischen Daten und der jeweiligen Wirtsbereiche nicht vorhanden ist, welche aber für potenzielle klinische Anwendungen notwendig wäre.

Anfang der 2000er-Jahre wurde am Leibniz-Institut DSMZ GmbH (Deutsche Sammlung von Mikroorganismen und Zellkulturen) in Braunschweig eine auf medizinisch relevante Bakterien spezialisierte Arbeitsgruppe und darüber hinaus das DZIF Stammrepositorium (zentrale Biobank des Deutschen Zentrums für Infektionsforschung) gegründet. Die daraus resultierende Phagenbank enthält deutlich mehr als 1000 Phagen für mehr als 150 Bakterienarten, wobei Phagen für die ESKAPE-Bakterien die Sammlung dominieren und in Forschungsprojekten eingesetzt werden. Der Schwerpunkt der DSMZ-Phagenbank^1^ wird durch den medizinischen Bedarf bestimmt. Eine direkte Anwendung am Menschen ist jedoch aus Gewährleistungsgründen ausgeschlossen. Für den genomischen Echtheitsnachweis der Phagen wurde dem DSMZ im Jahr 2022 von der zuständigen Behörde die Zertifizierung der GMP-Konformität nach § 64 Abs. 3f AMG erteilt.

Eine weitere große Phagenbank befindet sich am Institut für Mikrobiologie der Bundeswehr (IMB) in München [12]. Das Institut konzentriert sich derzeit auf Phagen, die spezifisch für *Burkholderia* spp., *Klebsiella* spp. und *Y. pestis* sind. Biowaffen-relevante Bakterienpathogene sind größtenteils noch empfindlich gegenüber einer Antibiotikatherapie, wurden jedoch im Rahmen eines sowjetischen Biowaffenprogramms vor 1991 genetisch verändert, um sie multiresistent gegen Antibiotika zu machen. Medizinische Gegenmaßnahmen in Form von Therapien auf der Basis von Phagen wären wahrscheinlich entscheidend für die Eindämmung von Infektionen mit diesen Pathogenen, wenn sie von einzelnen oder staatlichen Akteuren absichtlich freigesetzt würden. Das IMB erweitert derzeit seine Phagensammlung speziell für *K. pneumoniae* und trägt zu europäischen Phagendepots im Pirnay-Labor – Queen Astrid Military Hospital Brussels, Phage Directory sowie Citizen Phage, UK – bei.

Die Phagenforschung am Bundesinstitut für Risikobewertung (BfR, Berlin) basiert auf der grundlegenden Untersuchung des Vorkommens von Phagen, ihrer genetischen Vielfalt und ihres Anwendungspotenzials insbesondere im Veterinär- und Lebensmittelbereich. Daher werden die Kultursammlungen von lebensmittelbedingten Krankheitserregern sowie Bakterien, die im Rahmen des jährlichen Zoonose-Monitorings aus Tierbeständen gewonnen werden, für Phagentests verwendet. Die hierfür aufgebaute Phagensammlung gegen lebensmittelbedingte Krankheitserreger stammt hauptsächlich aus Proben der Lebensmittelproduktionskette (z. B. Prozesswasser von Schlachthöfen, Lebensmittelprodukten, Nutztieren), wurde aber auch aus Umweltquellen (z. B. Wildtieren) und/oder kommunalem/klinischem Abwasser gewonnen.

Eine weitere Forschungsgruppe in Erfurt (*HMU Health and Medical University*) und in Leipzig (Fraunhofer-Institut für Zelltherapie und Immunologie) verfügt über eine Phagenbank, die etwa 200 Phagen umfasst, die auf kritische Pathogene abzielen, darunter *K. pneumoniae, P. aeruginosa, S. aureus, A. baumannii *und *E. coli. *Darüber hinaus haben auch das Phage Center Regensburg (siehe oben) sowie das Zentralinstitut für Infektionsprävention (ZIP) der TU-München (siehe oben) eine nicht nur auf ESKAPE-Erreger abzielende Phagensammlung aufgebaut.

## Laufende Forschungsthemen und -projekte

„Phage4Cure“ (Charité Universitätsmedizin Berlin, Braunschweig, https://phage4cure.de/de/, gefördert durch das Bundesministerium für Bildung und Forschung – BMBF) ist eine First-in-Human-Studie (FIH) zur Untersuchung der Sicherheit, Verträglichkeit und vorläufigen Wirksamkeit eines Phagencocktails bei gesunden Probanden und Patienten mit chronischer Infektion der Lunge (Mukoviszidose) durch *P. aeruginosa*. Die in der klinischen Studie untersuchten 3 Phagen wurden mithilfe der Phagen-Biobank des Leibniz-Instituts DSMZ GmbH ausgewählt und am Fraunhofer-Institut (FH ITEM) gemäß den EU-GMP-Standards produziert. Der Cocktail wird als Aerosol über einen CE-zertifizierten Vernebler verabreicht. Wirksamkeit und Sicherheit wurden zuvor in Tiermodellen getestet. Die Phage4Cure-Studie besteht aus 2 Teilen: Teil 1 ist ein klassisches *Single-Ascending-Doses-*(SAD-)Design an gesunden Freiwilligen, Teil 2 ist ein *Multiple-Dose-Design*, das an Patienten mit chronischer *P.-aeruginosa*-Besiedlung der Lunge durchgeführt wird, bei denen zuvor nachgewiesen wurde, dass sie für diese spezielle Phagenkombination empfänglich sind.

„PhagoFlow“ (Bundeswehrkrankenhaus Berlin, Braunschweig, https://www.phagoflow.de/en/; gefördert durch den Innovationsfonds, Gemeinsamer Bundesausschuss – G-BA) ist ein Projekt, das untersucht, ob es mit den heutigen biopharmazeutischen Möglichkeiten möglich ist, Phagenpräparate in der Krankenhausapotheke individuell an den Patienten anzupassen und rechtzeitig für den therapeutischen Einsatz vorzubereiten – es wird somit die Praktikabilität der magistralen Herstellung von Phagenprodukten untersucht (keine klinische Studie im eigentlichen Sinne). Die Phagen wurden an der DSMZ isoliert und im FH ITEM produziert. Die Erreger werden aus dem Wundmaterial eines Patienten isoliert, die Phagenwirksamkeit wird untersucht (Phagogramm) und auf diesem Test basierend in der Krankenhausapotheke ein maßgeschneidertes Phagenpräparat hergestellt (individualisierte Therapie). Die Behandlung, zunächst mit Phagen gegen multiresistente *P. aeruginosa*, wurde im Februar 2023 begonnen.

„MAPVAP“ (präklinische Bewertung von 2 Phagencocktails, die auf multiresistente *P. aeruginosa* und *E. coli* abzielen, zur Behandlung von beatmungsassoziierter Lungenentzündung – VAP; [13]) ist ein kollaboratives binationales Forschungsprogramm zur Bekämpfung von Antibiotikaresistenzen (finanziert d. BMBF und Agence Nationale de la Recherche – ANR, Frankreich), in dem die Charité Universitätsmedizin Berlin die Co-Leitung inne hat. Mit 2 etablierten Phagencocktails, die spezifisch für *P. aeruginosa* oder *E. coli *sind, will MAPVAP u. a. die Entwicklung einer Phagenresistenz, die Auswirkungen dieser Cocktails auf die respiratorische und intestinale Mikrobiota, die Wirksamkeit der Cocktails bei der Durchdringung von Biofilmen und die direkte Interaktion von Phagen mit dem Immunsystem bewerten [13].

„IDEAL-EC“ (Universitätsklinikum Köln) konzentriert sich in Zusammenarbeit mit dem Leibniz-Institut DSMZ und dem DZIF auf die Evaluierung der Wirkung von Phagen gegen Extended-Spectrum-Beta-Lactamase-produzierende klinische *E.-coli*-Proben. Ein *E.-coli*-Phagencocktail wird hierbei in einem präklinischen Modell (*In-vitro*-Darmmodell, Mausmodell) untersucht.

„EVREA-Phage“ (DSMZ GmbH, Braunschweig) ist ein vom DZIF gefördertes, translationales präklinisches Projekt mit dem Ziel, neue Phagen zu charakterisieren und einen Phagencocktail gegen *E. faecium* für eine spätere orale Anwendung als Option zur spezifischen intestinalen Dekolonisierung von Vancomycin-resistenten Enterokokken (VRE) *E. faecium* bei immungeschwächten Patienten zusammenzustellen. Für die vollständige biologische Charakterisierung der Phagen wurden umfangreiche Phagen- und Stamm-Panels zusammengestellt. Bei den Stämmen handelt es sich um klinische Isolate aus deutschen Universitätskliniken, die dem DZIF-Netzwerk angehören, und aus dem Robert Koch-Institut. Alle Phagen wurden vom Leibniz-Institut DSMZ neu isoliert. EVREA-Phage sieht vor, die Wirksamkeit der Phagen in *In-vitro-* und *In-vivo*-Darmmodellen am Universitätsklinikum Bonn und am Helmholtz-Zentrum für Infektionsforschung, Braunschweig, zu demonstrieren.

„Phage2030“ (Bundeswehrkrankenhaus Berlin) hatte das Ziel, realisierbare Forschungsanstrengungen und Herstellungswege zu identifizieren, deren Koordination und gezielte Förderung einen „Sprung nach vorn“ bedeuten würde, um die Phagentherapie bis 2030 der deutschen Öffentlichkeit zugänglich zu machen. Die dafür notwendige Validierungsstudie wurde von der Bundesagentur für disruptive Innovationen SPRIN‑D gefördert.^2^

„CRAB-A-Phage“ (Universitätsklinikum Köln) ist ein weiteres Projekt in enger Zusammenarbeit zwischen dem Leibniz-Institut DSMZ und dem DZIF. Es konzentriert sich auf die präklinische Evaluierung von Phagen-Antibiotika-Synergien bei multiresistenten *A. baumannii*.

Am Helmholtz-Zentrum *München* und an der Technischen Universität München (TUM) werden mehrere Projekte durchgeführt, die durch das *Innovative Training Network* (ITN; „VIROINF“) des Europäischen Forschungsrats, das DFG-Schwerpunktprogramm „Novel Concepts in Phage Biology“ und das Sequenzierprogramm „Asthma-Phage“ finanziert werden und die darauf abzielen, die Wechselwirkungen zwischen dem Virom, dem Mikrobiom und dem menschlichen Wirt bei Dysbiose-assoziierten Verdauungs- und Atemwegserkrankungen (z. B. Asthma) zu erforschen und die verursachenden Bakterien mithilfe synthetischer Phagengemeinschaften auszurotten (Phageomtherapie). Ein weiteres Projekt ist „Phagentherapie gegen Darmkrebs-assoziierte Helicobacter- und NEC-Erreger“ (DFG-gefördert). Zudem wird derzeit eine Hochdurchsatz-Pipeline zur automatischen Isolierung und Charakterisierung von Phagen sowie eine gut charakterisierte Phagenbank gegen hochkritische Bakterien, einschließlich ESKAPE-Erregern, aufgebaut. Ebenso werden optimierte Phagencocktails in klinisch relevanten Schweinemodellen getestet. Darüber hinaus zielt „PHARMS“ (finanziert durch *ERC Starting Grant*) darauf ab, neue antibakterielle Wirkstoffe gegen *A. baumannii, Helicobacter pylori* und *Haemophilus influenzae* zu entdecken, indem Phagen-Wirt-Interaktionen mit kulturunabhängigen und Multi-Omik-Ansätzen untersucht werden. „COVPHA“ (BMBF-gefördert) zielt darauf ab, die koinfizierenden multiresistenten Bakterien bei COVID-19-Patienten durch Metagenomik zu identifizieren und wirksame Phagen gegen sie zu isolieren.

In enger Zusammenarbeit der Universitätskliniken Köln und Frankfurt am Main werden zahlreiche translationale Forschungsprojekte zur Phagentherapie vorangetrieben mit dem Ziel, noch 2025 eine personalisierte Anwendung von GMP-Phagen für Therapien zu ermöglichen. Die Entwicklung der AWMF-S2k-Leitlinie zur personalisierten Phagentherapie wurde ebenfalls hier koordiniert (s. oben).

Die Therapeutische Phagengruppe des Instituts für Mikrobiologie der Bundeswehr (IMB) in München arbeitet mit der Europäischen Kommission zusammen. In diesem Netzwerk werden *Klebsiella*-Phagen und ihre bakteriellen Wirte charakterisiert, optimiert und für therapeutische Anwendungen mit einer One-Health-Perspektive vorbereitet (Joint Programming Initiative on Antimicrobial Resistance – JPIAMR, gefördertes KLEOPATRA-Konsortium^3^). Das IMB ist ebenfalls aktiv im Forschungskonsortium des Europäischen Verteidigungsfonds (EDF) „RESILIENCE-R-2023 Project“. Ziel der eigenen Projekte ist es, Experimente zur Erweiterung des Wirtsspektrums von Phagen zu entwerfen, die azelluläre Methode zur Herstellung therapeutischer Phagen zu optimieren, die Phagentherapie in klinischen Studien und individuellen Heilungsversuchsprotokollen mit personalisierten Phagenpräparaten zu unterstützen und die therapeutischen Ansätze der klinischen Partner zu verbessern [14]. Ebenso gehören zu den methodischen Aspekten der Phagenarbeit am IMB die Phagen-Antibiotika-Synergie (PAS), Rezeptor‑/Virulenzfaktor-Bindungsproteine und die Erprobung therapeutischer Phagenprotokolle im Modell der Großen Wachsmotte (Galleria mellonella).

Die wichtigsten Bemühungen der Forschungsgruppe in Erfurt (*HMU Health and Medical University*) und in Leipzig (Fraunhofer-Institut für Zelltherapie und Immunologie) sind Genomanalysen neuartiger Phagenisolate, um sicherzustellen, dass keine Gene für antimikrobielle Resistenzen, Virulenzfaktoren oder Gene, die auf Lysogenie hinweisen, vorhanden sind. Darüber hinaus werden Gene identifiziert, die für Proteine mit antibakteriellen und antibiofilmen Eigenschaften kodieren könnten, um das Potenzial von aus Phagen gewonnenen Therapeutika zur Bekämpfung von antibiotikaresistenten Infektionen oder solchen mit Biofilmen zu erforschen. Da die Phagentherapie als „personalisierte Medizin“ angesehen werden kann, werden Pipelines für die Phagenproduktion und -reinigung für einzelne Patienten entwickelt.

## Fazit und Ausblick

Ziel war es, die aktuelle Landschaft der Phagentherapie in Deutschland aus der Sicht des Klinikers darzustellen. Da Phagentherapeutika derzeit klinisch nicht zugelassen sind, wird die Phagentherapie nur von Universitätskliniken und einem Bundeswehrkrankenhaus ausnahmslos unter dem Dach des Artikels 37 der Deklaration von Helsinki bei einer begrenzten Anzahl von Patienten angewendet. In der Regel werden hierfür Phagenkompositprodukte (Cocktails) verwendet, die typischerweise gebrauchsfertig aus dem Ausland bezogen oder im Rahmen der erlaubnisfreien Herstellung im jeweiligen Krankenhaus für die eigenen Patienten unter Aufsicht des zuständigen Arztes hergestellt werden. Die bisherigen Einschränkungen führen dazu, dass in der unmittelbaren Vergangenheit jährlich weniger als 100 Patienten in Deutschland behandelt wurden. Die bisher in Deutschland bestehenden Phagenbanken arbeiten aufgrund ihres unterschiedlichen institutionellen Hintergrunds nicht in vergleichbarer Weise und tauschen ihre Phagenbestände i. d. R. nur für wissenschaftliche Projekte aus. Die Phagenproduktion erfolgte bisher nur an ganz wenigen Standorten und i. d. R. nur für die eigenen Patienten.

Viele Phagengruppen, die Grundlagenforschung betreiben, wurden im vorliegenden Artikel nicht genannt, sie finden sich jedoch zusammengestellt unter [2]. Es werden neben translationalen Ansätzen, z. B. zur Erleichterung des Bakterien-Phagen-Matching-Prozesses [15] oder zur Nutzung von Phagen-Antibiotika-Synergien [16], auch umfassende Schwerpunktprogramme (z. B. „New Concepts in Prokaryotic Virus-host Interactions – From Single Cells to Microbial Communities“ der DFG; https://spp2330.de/) verfolgt. Obwohl die Finanzierungssituation und das Bewusstsein weiter verbessert werden müssen, wurden wichtige Schritte unternommen, um die Möglichkeit zu nutzen, die Herausforderung durch multiresistente Erreger (MRE) mit dem Einsatz der Phagentherapie anzugehen. Hervorzuheben ist, dass bereits höhere Summen öffentlicher Mittel für Forschungsprojekte eingesetzt wurden, was ebenso wie das vom Deutschen Bundestag 2021 in Auftrag gegebene Gutachten zur Phagentherapie [17] das ernsthafte Interesse der politischen Entscheidungsträger unterstreicht.

Nach Ansicht der Autoren sollten mit dem Nachweis der klinischen Wirksamkeit von Phagentherapeutika die derzeitigen rechtlichen Rahmenbedingungen für die Verwendung und Herstellung von Phagen an die besonderen Anforderungen dieser Therapeutika angepasst werden, um einen zeitnahen Zugang für Patienten zu gewährleisten. Hierfür sind die kontrollierte, qualitativ hochwertige Phagenproduktion und -therapie von großer Bedeutung. Optionen wie das belgische Modell für Herstellung und Anwendung sollten in Betracht gezogen und rechtlich verankert werden.

Die aktuelle Situation zeigt eine von großem Enthusiasmus getragene Aktivität, das Potenzial zu erforschen und die Phagentherapie in Deutschland möglichst schnell umzusetzen. Es besteht die Notwendigkeit, mehr Phagen für die klinische Erprobung und Anwendung zur Verfügung zu stellen. Die Produktionskapazitäten müssen ausgebaut sowie Qualitätsstandards für Phagenproduktion und klinische Anwendung von Phagenprodukten entwickelt werden, damit der klinische Einsatz von Phagenprodukten in Deutschland einen entscheidenden Schritt im Kampf gegen multiresistente Bakterien darstellen wird.

### Infobox „DZIF Translational Phage-Netzwerk“

Das translationale Phagennetzwerk des Deutschen Zentrums für Infektionsforschung (DZIF), sog. DZIF Translational Phage-Netzwerk (kurz: DZIF TransPhage-Net), hat es sich zur Aufgabe gemacht, Phageninteressierte zusammenzubringen und den Austausch mit Pharma-Produzierenden und regulatorischen Behörden zu verbessern. Auch internationale Expertengremien auf dem Gebiet der Phagenforschung sind in dem Netzwerk willkommen, um weitere Impulse einbringen zu können. Durch das Netzwerk soll eine bestmögliche Implementierung der Phagenforschung, -entwicklung und -therapie in Deutschland vorangetrieben werden. Regelmäßige virtuelle und persönliche Treffen fördern die Netzwerkbildung und den Austausch über aktuelle Forschungs- und Behandlungsansätze (https://www.dzif.de/de/dzif-transphage-net).

## Footnotes

1 Siehe auch Collection Experts: https://www.dsmz.de/de/collection/collection-experts; zugegriffen: 18.03.2025

2 SPRIN-D-Abschlussbericht unter https://www.sprind.org/magazin/phage2030; zugegriffen: 18.03.2025.

3 KLEOPATRA (Design and implementation of effective cOmbination of Phages and Antibiotics for improved TheRApy protocols against KLEbsiella pneumoniae); Projekt-Webseite: https://www.jpiamr.eu/projects/kleopatra/; zugegriffen: 18.03.2025.
